# Association between thyroid hormones and the components of metabolic syndrome

**DOI:** 10.1186/s12902-018-0256-0

**Published:** 2018-05-21

**Authors:** Jieun Jang, Youngsook Kim, Jaeyong Shin, Sang Ah Lee, Young Choi, Eun-Cheol Park

**Affiliations:** 1 Department of Public Health, Graduate School, Yonsei University, Seoul, Republic of Korea; 2 Institute of Health Services Research, Yonsei University, Seoul, Republic of Korea; 3 Department of Anesthesia, Indiana University School of Medicine, Indianapolis, 46202 USA; 4 Department of Preventive Medicine & Institute of Health Services Research, Yonsei University College of Medicine, 50 Yonsei-ro, Seodaemun-gu, Seoul, 120-752 Republic of Korea

**Keywords:** Thyroid hormone, Metabolic syndrome, Free thyroxine, FT4

## Abstract

**Background:**

Thyroid hormones are known to have direct and indirect effects on metabolism. Individuals with metabolic syndrome, a disease that is growing in incidence at a rapid rate, are at higher risk for cardiovascular disease, diabetes, and cancer. The aim of this study was to identify whether significant correlations exist between thyroid hormone levels and components of the metabolic syndrome in the general population of Korea.

**Methods:**

The data were collected from the sixth Korea National Health and Nutrition Examination Surveys from 2013 to 2015. A total of 1423 participants were tested for thyroid function. The analysis of variance and multiple linear regression were performed to analyze the relationship between thyroid hormone level and components of the metabolic syndrome.

**Results:**

A positive association between free thyroxine and fasting glucose level was observed in patients with high free thyroxine levels (≥1.70 ng/dL, β = 15.992, *p* = < 0.0001), when compared with patients with normal-middle free thyroxine levels. Moreover, a negative association was observed between free thyroxine and triglyceride levels in patients with normal-high free thyroxine levels (β = − 21.145, *p* = 0.0054) and those with high free thyroxine levels (β = − 49.713, *p* = 0.0404).

**Conclusion:**

Free thyroxine shows a partially positive association with fasting glucose and a partially negative association with triglycerides in the Korean population. In patients with abnormal thyroid function, follow up tests for glucose levels and lipid profiling during treatment for thyroid dysfunction would be beneficial in terms of overlooking metabolic syndrome and to prevent related diseases.

**Electronic supplementary material:**

The online version of this article (10.1186/s12902-018-0256-0) contains supplementary material, which is available to authorized users.

## Background

Metabolic syndrome, a well-known cluster of cardiovascular risk factors, is a major public health concern worldwide [1, 2]. Metabolic syndrome increases the risk for cardiovascular disease, diabetes, and even certain types of cancer [3]. According to the National Cholesterol Education Program’s Adult Treatment Panel III definition, metabolic syndrome is the presence of abnormal values for at least three of the following criteria: waist circumference, serum triglycerides, high-density lipoprotein (HDL) cholesterol, blood pressure, and fasting glucose [3]. The prevalence of metabolic syndrome is increasing rapidly [4]. According to data from the National Health and Nutrition Examination Survey (NHANES) 2011–2012, about 34.7% of US adults were estimated to have metabolic syndrome [4]. Similar increasing trends have been observed in Europe and other countries [2, 5]. According to the National Cholesterol Education Program’s Adult Treatment Panel III criteria, and per the World Health Organization Asia-Pacific guidelines, the prevalence of metabolic syndrome was approximately 28.2% in the general population of Korea in 2012 [6]. The mortality rate due to cardiovascular disease has increased from 35.6 to 52.4 out of 100,000 persons over 2003–2014. Thus, more focus and effort is needed to reduce the prevalence of metabolic syndrome, while considering the rapidly increasing frequency of mortality due to related diseases [7].

Thyroid hormones have an important role in metabolism [8]. Abnormal levels of thyroid hormones alter metabolism, and some of these changes share common pathophysiologic processes with metabolic syndrome. Therefore, thyroid dysfunction can affect metabolic syndrome. Lambadiari et al. reported that thyroid hormones are significant determinants of glucose homeostasis [9], and affect fasting glucose levels by antagonizing insulin action [9]. For instance, hyperthyroidism leads to impaired insulin secretion, which suppresses hepatic glucose production and promotes glucose uptake in the muscle [9]. Similarly, Dimitriadis et al. showed that the increased glucose levels in hyperthyroidism could be explained by an increase in endogenous glucose production via of gluconeogenesis [10]. Studies have also demonstrated an association between thyroid hormones and glucose levels. Klein et al. reviewed several studies regarding the mechanism of action of thyroid hormones on the cardiovascular system [11]. They concluded that thyroid hormones have direct and indirect impacts on the cardiovascular system. Patients with thyroid disease, especially hyperthyroidism, often demonstrated signs and symptoms of cardiovascular changes [11]. Several other studies have shown that overt hypothyroidism induces an increase in blood pressure and plasma cholesterol levels [12]. However, most of the studies that assessed the relationships between abnormal thyroid hormone levels and metabolic syndrome have been conducted in Caucasian populations [2, 13]. Therefore, the aim of this study was to assess the relationships between thyroid hormone levels and metabolic syndrome components in a nationally representative sample of South Korean adults.

## Methods

### Study population

This study was conducted using data from the sixth Korea National Health and Nutrition Examination Surveys (KNHANES VI, 2013–2015), a nationwide cross-sectional survey conducted by the Korean Centers for Disease Control and Prevention (Seoul, Korea) to assess the health and nutritional status of the South Korean population. The institutional review board of the Korea Centers for Disease Control and Prevention (KCDC) approved the study (IRB: 2013-07CON-03-4C, 2013-12EXP-03-5C, 2015–01-02-6C). A nationally representative sample was obtained using a stratified multistage cluster sampling design. The survey consists of a health interview, nutrition survey, and health examination. Tests for thyroid disease and metabolic syndrome components (waist circumference, triglycerides, HDL cholesterol, blood pressure, and fasting glucose) were part of the health examination. The initial sample included 22,948 participants. The thyroid functions tests were carried out by subsampling 2400 subjects aged ≥10 years with respect to thyroid-stimulating hormone (TSH), free thyroxine (FT4), thyroid peroxidase antibody (TPOab), and urinary iodine in the sixth Korea National Health and Nutrition Examination Surveys (KNHANES VI, 2013–2015). Participants with missing thyroid function data were excluded (*n* = 20,591). Patients who received treatment that could interfere with the test results for thyroid hormone levels and various components of metabolic syndrome, such as radioactive iodine treatment, antithyroid drugs, thyroid hormones, other medication for thyroid disease, dyslipidemia medications, blood pressure regulators, insulin, or glucose regulators were also excluded (*n* = 429). The data for a total of 1423 participants without any missing variables were finally included in the analysis.

### Dependent variables and variables of interest

The dependent variables were the components of metabolic syndrome. Waist circumference was measured at the narrowest spot between the lowest rib and the highest lateral border of the right iliac crest. Systolic blood pressure was measured after the participants relaxed for 5 min while sitting. Triplicate measurements of systolic blood pressure were obtained. The mean of the second and third measured value was used in the analysis. Triglycerides, HDL cholesterol, and fasting glucose levels were measured using the same Hitachi Automatic Analyzer 7600–210 (Hitachi, Tokyo, Japan).

The variable of interest in this study was thyroid hormone levels. Serum FT4, serum TSH, and TPOab levels were measured using an electro-chemiluminescence immunoassay (Cobas: Roche Diagnostics, Penzberg, Germany). Samples were sent to the central certified laboratory and analyzed. The laboratory reference ranges for FT4, TSH, and TPOab were 0.80–1.70 ng/dL, 0.50–5.0 uIU/mL, and < 34 IU/mL [14–16]. FT4 levels were divided into five categories: (1) low; under normal (< 0.80 ng/dL); (2) normal-low (< 1.17 ng/dL); (3) normal-middle (< 1.31 ng/dL); (4) normal-high (< 1.70 ng/dL); and (5) high; upper normal (≥1.70 ng/dL). TSH levels were divided as follows: low; under normal (< 0.50 uIU/mL); normal-low (< 1.80 uIU/mL), normal-middle (< 2.87 uIU/mL), normal-high (< 5.00 uIU/mL) and high; upper normal (≥5.00 uIU/mL). TPOab levels were categorized as normal (< 34 IU/mL), high with low FT4 (34 IU/mL ≤ TPOab; FT4 < 1.24 ng/dL), high with high FT4 (34 IU/mL ≤ TPOab; 1.24 ng/dL ≤ FT4). In addition, we categorized TPOab levels into normal (< 34 IU/mL), high with low TSH (34 IU/mL ≤ TPOab; TSH < 2.25 uIU/mL), high with high TSH (34 IU/mL ≤ TPOab; 2.25 uIU/mL ≤ FT4). Considering that the TPOab titer is related to both hypothyroidism and hyperthyroidism, patients with high levels were divided into two groups using two median FT4 levels and two TSH levels.

### Covariates

We adjusted for the covariates of sociodemographic factors, socioeconomic factors, health-behavior factors, and health-condition factors. Sociodemographic factors included age (19–44 years, 45–64 years, > 64 years) and gender (male and female). Socioeconomic factors included educational level (elementary school or less, middle school, high school, and college or over), marital status (married and cohabit, married but no cohabit or bereaved or divorced, and unmarried), household income level (divided into quartiles), region (urban or rural), and occupation (white collar, pink collar, blue collar, and unemployed or else). Urban areas included capitals and metropolitan cities and rural areas comprised the remaining areas. Alcohol consumption (ever or never), smoking (ever or never), and walking activity (active or inactive) were the health-behavior factors. Health-condition factors involved stress level (high, middle, low).

### Statistical analysis

Statistical analysis was performed using the SAS software, version 9.4 (SAS Institute, Cary, NC, USA). All analysis incorporated weights. The mean values and standard deviations of the components of metabolic syndrome were compared using the analysis of variance. Multiple linear regression was performed to analyze the relationship between thyroid hormone level and the components of metabolic syndrome. Subgroup analysis was performed according to age and gender. A *p*-value < 0.05 was considered to indicate a statistically significant result.

## Results

### Demographic characteristics

Table 1 presents the general characteristics of the study populations. There were 683 males (48.0%) and 740 females (52.0%) included in this study. Of the 1423 participants, 870 (61.1%) were aged between 19 and 44 years, 471 participants (33.1%) were aged between 45 and 64 years, and 82 participants (5.8%) were aged over 64 years. The mean waist circumference was 31.9 ± 4.0 in. and the mean triglycerides level was 133.0 ± 120.5 mg/dL. The mean HDL cholesterol level was 52.0 ± 12.8 mg/dL, mean blood pressure 114.2 ± 15.1 mmHg, and the mean fasting glucose level 95.0 ± 16.6 mg/dL. When analyzed according to FT4 levels, waist circumference, triglycerides, and fasting glucose results were statistically significantly different (*p* < 0.05). Patients with high FT4 levels demonstrated considerably higher fasting glucose levels (High: 109.4 ± 45.2 mg/dL; Normal-middle: 93.8 ± 12.2 mg/dL).

**Table 1:** General characteristics of the study population

	Waist circumference (inches)					Triglycerides (mg/dL)			HDL cholesterol (mg/dL)			Blood pressure (mmHg)			Fasting glucose (mg/dL)		
	*N*	%	Mean ± SD		*p*-value	Mean ± SD		*p*-value	Mean ± SD		*p*-value	Mean ± SD		*p*-value	Mean ± SD		*p*-value
Total	1423	100.0	31.9	4.0		133.0	120.5		52.0	12.8		114.2	15.1		95.0	16.6	
Free thyroxine hormone (ng/dL)					0.0388			0.0020			0.1873			0.9267			<.0001
Low (< 0.80)	15	1.1	32.8	2.4		162.7	74.4		51.8	13.4		116.3	12.5		97.1	14.4	
Normal (low tertile) (< 1.17)	424	29.8	32.1	4.1		141.3	131.9		51.8	13.1		114.6	16.0		95.9	16.8	
Normal (mid tertile) (< 1.31)	488	34.3	31.8	3.9		133.9	118.3		52.1	12.2		113.9	15.6		93.8	12.2	
Normal (high tertile) (< 1.70)	472	33.2	31.9	4.2		125.2	114.9		52.2	13.2		113.9	13.9		94.5	17.5	
High (1.70≤)	24	1.7	31.8	4.1		102.1	53.0		48.2	7.2		116.6	11.8		109.4	45.2	
Thyroid stimulating hormone (uIU/mL)					0.2965			0.9867			0.1489			0.6965			0.1958
Low (< 0.50)	30	2.1	30.8	2.9		108.4	66.1		55.9	14.5		116.3	14.2		94.9	10.9	
Normal (low tertile) (< 1.80)	476	33.5	32.1	4.1		136.6	144.8		51.3	13.1		114.3	14.9		96.2	19.4	
Normal (mid tertile) (< 2.87)	468	32.9	32.1	4.0		131.7	111.9		52.1	12.5		113.6	14.8		94.6	13.4	
Normal (high tertile) (< 5.00)	334	23.5	31.6	4.1		132.0	104.4		52.5	12.7		114.7	16.0		94.8	18.4	
High (5.00≤)	115	8.1	31.7	4.0		132.9	96.0		51.6	12.3		114.0	14.7		92.1	10.8	
Thyroid peroxidase antibody (IU/mL)					0.5217			0.5189			0.4016			0.0369			0.9543
Normal (< 34)	1324	93.0	31.9	4.1		132.2	120.2		52.0	12.8		114.0	15.1		95.1	17.0	
High (under low FT4) (34≤)	56	3.9	31.7	4.0		155.3	149.2		50.6	12.5		114.4	13.1		92.6	10.1	
High (under high FT4) (34≤)	43	3.0	31.5	3.1		127.9	79.6		51.5	13.7		119.4	16.6		94.0	10.6	
Thyroid peroxidase antibody (IU/mL)					0.9967			0.3755			0.5473			0.0827			0.5928
Normal (< 34)	1324	93.0	31.9	4.1		132.2	120.2		52.0	12.8		114.0	15.1		95.1	17.0	
High (under low TSH) (34≤)	39	2.7	31.5	4.2		124.8	69.9		50.5	12.5		114.8	14.3		93.7	12.0	
High (under high TSH) (34≤)	60	4.2	31.6	3.3		155.5	148.5		51.3	13.4		117.7	15.2		93.0	9.1	
Age					0.4481			0.0347			0.0217			<.0001			0.0746
19–44	870	61.1	31.5	4.3		125.1	119.9		53.5	12.7		109.9	12.2		92.9	16.7	
45–64	471	33.1	32.6	3.4		149.0	126.9		50.0	13.0		119.9	16.6		98.1	15.4	
64<	82	5.8	32.9	3.4		125.4	69.6		47.5	9.8		126.1	16.8		98.3	19.3	
Gender					<.0001			<.0001			<.0001			<.0001			<.0001
Male	683	48.0	33.7	3.8		166.2	151.9		47.7	11.1		118.4	14.2		98.0	20.1	
Female	740	52.0	30.3	3.6		102.4	68.3		55.9	13.0		110.3	14.9		92.2	12.0	
Education level					0.7844			0.5789			0.0002			0.0034			0.9794
Elementary school or less	119	8.4	32.8	3.1		150.5	114.0		46.3	11.0		124.4	15.0		97.6	12.0	
Middle school	108	7.6	32.6	4.2		148.5	105.8		50.6	11.6		120.8	15.9		97.4	15.2	
High school	574	40.3	31.9	4.0		132.5	126.4		53.0	13.2		114.5	15.4		94.9	16.3	
College or over	622	43.7	31.6	4.2		127.4	118.2		52.3	12.7		110.8	13.3		94.1	17.8	
Marital status					<.0001			0.5017			0.0569			0.2066			<.0001
Married-cohabit	813	57.1	32.3	3.7		136.4	110.3		51.3	12.4		116.0	16.3		96.7	17.9	
Married-no co habit or bereaved or divorced	130	9.1	32.5	3.8		147.0	121.8		50.0	13.5		118.0	15.5		98.8	18.4	
Unmarried	480	33.7	31.1	4.5		123.5	135.2		53.8	12.9		110.1	11.5		91.0	12.8	
Household income level					0.0328			0.6671			0.1671			0.0484			0.4857
Quartile 1 (lowest)	346	24.3	32.5	4.3		137.7	125.9		51.3	13.0		116.5	16.3		95.2	15.3	
Quartile 2	336	23.6	31.8	3.8		124.2	100.7		52.5	13.2		113.5	14.2		94.8	18.1	
Quartile 3	386	27.1	31.9	3.8		137.2	115.6		51.0	12.4		113.9	14.5		94.3	11.8	
Quartile 4 (highest)	355	24.9	31.5	4.1		132.2	136.4		53.1	12.6		112.7	15.1		95.6	20.5	
Region					0.0163			0.0112			0.3290			0.6709			0.7321
Urban area	1043	73.3	31.7	3.9		127.4	113.4		52.3	12.6		113.6	15.1		94.6	16.4	
Rural area	380	26.7	32.5	4.3		148.5	137.1		51.1	13.2		115.9	14.9		96.0	17.2	
Occupation					0.1036			0.7295			<.0001			0.5925			0.1260
White color	445	31.3	31.9	4.1		132.0	117.5		51.7	13.2		111.4	13.5		94.9	19.3	
Pink color	209	14.7	32.0	3.9		126.2	95.8		52.9	12.5		114.1	14.1		94.6	13.6	
Blue color	315	22.1	33.0	3.6		157.2	147.9		51.4	13.3		120.1	15.9		98.6	17.6	
Unemployed or else	454	31.9	31.2	4.2		120.3	109.8		52.2	12.1		112.9	15.4		92.7	13.8	
Alcohol consumption					0.0108			0.3359			0.2579			0.8845			0.4812
Ever	85	6.0	32.1	3.7		123.2	95.3		51.5	13.9		116.0	17.3		95.5	11.2	
Never	1338	94.0	31.9	4.1		133.6	121.9		52.0	12.7		114.1	14.9		94.9	16.9	
Smoking					0.0062			0.0025			0.5679			0.6979			0.3499
Ever	603	42.4	33.5	4.1		167.2	146.6		48.8	12.8		117.5	15.0		97.2	16.3	
Never	820	57.6	30.8	3.6		107.9	88.9		54.2	12.3		111.7	14.7		93.3	16.7	
Walking activity					0.8915			0.3812			0.4642			0.0845			0.1128
Active	581	40.8	31.8	3.9		127.3	121.2		52.5	12.7		112.7	14.3		93.5	13.8	
Inactive	842	59.2	32.0	4.1		137.0	119.8		51.6	12.8		115.2	15.5		96.0	18.3	
Stress level					0.1867			0.0485			0.0567			0.1300			0.5092
High	451	31.7	31.9	4.4		141.3	145.7		51.9	12.6		112.6	14.0		95.0	18.7	
Middle	800	56.2	31.8	3.9		128.1	101.0		52.0	12.9		114.0	15.1		94.7	14.9	
Low	172	12.1	32.6	3.7		134.0	129.7		52.1	12.9		119.1	16.9		96.2	18.5	

### Multiple analysis

Table 2 shows the estimates for the components of metabolic syndrome. “β” presents standardized regression coefficient and “S.E” presents standardized error of a correlation coefficient. After controlling for covariates, the results showed a positive association between FT4 and fasting glucose level in patients with high FT4 levels when compared to those with normal-middle FT4 levels (β = 15.992; *p =* < 0.0001). We also identified a significant negative association between FT4 levels and triglycerides in patients with normal-high (β = − 21.145, *p* = 0.0054) and those with high FT4 levels (β = − 49.713, *p* = 0.0404). In addition, a positive association was observed between TPOab and triglycerides levels in patients with high TPOab levels in the presence of low median FT4 levels (β = 36.075, *p* = 0.0247). Also, positive association was identified between TPOab and triglycerides levels in patients with high TPOab levels in the presence of high median TSH levels (β = 32.181, *p* = 0.0368).

**Table 2:** The estimates for the components of metabolic syndromes^a^

Variable	Waist circumference (inches)			Triglycerides (mg/dL)			HDL cholesterol (mg/dL)			Blood pressure (mmHg)			Fasting glucose (mg/dL)		
	β^a^	S.E	*p*-value	β^a^	S.E	*p*-value	β^a^	S.E	*p*-value	β^a^	S.E	*p*-value	β^a^	S.E	*p*-value
Free thyroxine hormone (ng/dL)															
Low (< 0.80)	1.383	0.938	0.1407	38.690	30.172	0.2000	−0.267	3.109	0.9317	0.758	3.532	0.8301	2.727	4.217	0.5180
Normal (low tertile) (< 1.17)	0.327	0.240	0.1731	9.957	7.705	0.1964	−0.279	0.801	0.7272	−0.712	0.905	0.4313	1.698	1.077	0.1152
Normal (mid tertile) (< 1.31)	Ref.			Ref.			Ref.			Ref.			Ref.		
Normal (high tertile) (< 1.70)	− 0.426	0.236	0.0714	−21.145	7.591	0.0054	1.439	0.788	0.0681	−0.359	0.890	0.6869	0.268	1.061	0.8003
High (1.70≤)	−0.735	0.753	0.3292	−49.713	24.227	0.0404	−1.718	2.603	0.5095	2.068	2.836	0.4661	15.992	3.386	<.0001
Thyroid stimulating hormone (uIU/mL)															
Low (< 0.50)	−1.253	0.673	0.0629	−20.614	21.742	0.3432	4.393	2.227	0.0487	2.656	2.569	0.3014	0.178	3.040	0.9533
Normal (low tertile) (< 1.80)	−0.318	0.234	0.1742	0.479	7.559	0.9495	−0.558	0.783	0.4759	0.630	0.880	0.4743	1.680	1.057	0.1122
Normal (mid tertile) (< 2.87)	Ref.			Ref.			Ref.			Ref.			Ref.		
Normal (high tertile) (< 5.00)	− 0.378	0.257	0.1414	3.591	8.297	0.6652	0.035	0.859	0.9671	1.283	0.966	0.1843	0.713	1.160	0.5392
High (5.00≤)	−0.093	0.374	0.8044	9.045	12.095	0.4547	−0.890	1.246	0.4748	0.660	1.406	0.6389	−1.511	1.691	0.3717
Thyroid peroxidase antibody (IU/mL)^b^															
Normal (< 34)	Ref.			Ref.			Ref.			Ref.			Ref.		
High (under low FT4) (34≤)	0.396	0.498	0.4268	36.075	16.046	0.0247	−2.475	1.674	0.1396	−0.814	1.866	0.6626	−2.152	2.249	0.3387
High (under high FT4) (34≤)	−0.620	0.556	0.2655	−6.054	17.922	0.7356	0.118	1.862	0.9493	4.319	2.084	0.0384	−1.604	2.512	0.5232
Thyroid peroxidase antibody (IU/mL)^c^															
Normal (< 34)	Ref.			Ref.			Ref.			Ref.			Ref.		
High (under low TSH) (34≤)	−0.307	0.587	0.6014	−5.248	18.895	0.7812	−1.617	1.966	0.4110	−0.493	2.198	0.8225	−1.511	2.648	0.5682
High (under high TSH) (34≤)	0.111	0.478	0.8162	32.181	15.396	0.0368	−1.130	1.608	0.4825	2.723	1.791	0.1288	−2.169	2.157	0.3149

^a^Models is adjusted by age, gender, education level, marital status, household income level, region, occupation, alcohol consumption, smoking, walking activity and stress level

^b^Considering that thyroid peroxidase antibody related to both hypothyroidism and hyperthyroidism, high groups were divided into two groups by two median of free thyroxine

^c^Considering that thyroid peroxidase antibody related to both hypothyroidism and hyperthyroidism, high groups were divided into two groups by two median of thyroid stimulating hormone

### Sensitivity analysis

Table 3 shows the subgroup analysis of the association between the components of metabolic syndrome and FT4 hormone levels according to age and gender. When stratified by age, a significant negative association between FT4 and triglycerides was observed among patients aged 19–44 years (low: β = 124.396, *p* = 0.0165; normal-high: β = − 25.519, *p* = 0.0050). This association was stronger in the 19–44-year-old age group than in the other older age groups.

**Table 3:** Subgroup analysis of components of metabolic syndromes with free thyroxine hormone levels stratified by age and gender

Variables	Free thyroxine hormone levels												
	Low			Normal (low tertile)			Normal (mid tertile)	Normal (high tertile)			High		
	β^a^	S.E	*p*-value	β^a^	S.E	*p*-value	β^a^	β^a^	S.E	*p*-value	β^a^	S.E	*p*-value
Waist circumference													
Age													
19~ 44	3.248	1.715	0.0586	0.400	0.345	0.2471	Ref.	−0.537	0.300	0.0738	−1.062	0.892	0.2343
45~ 64	0.745	1.113	0.5037	0.470	0.352	0.1818	Ref.	0.078	0.410	0.8495	0.394	1.668	0.8134
64<	−1.085	3.667	0.7684	−0.026	0.927	0.9777	Ref.	−1.638	1.226	0.1868	−3.990	3.705	0.2859
Gender													
Male	1.998	2.172	0.3580	0.804	0.405	0.0477	Ref.	−0.270	0.336	0.4206	−0.684	0.957	0.4749
Female	1.481	0.982	0.1322	0.070	0.284	0.8061	Ref.	−0.556	0.328	0.0909	−2.002	1.277	0.1173
Triglycerides													
Age													
19~ 44	124.396	51.798	0.0165	10.341	10.430	0.3217	Ref.	−25.519	9.066	0.0050	−49.843	26.938	0.0646
45~ 64	22.880	41.955	0.5858	14.699	13.253	0.2680	Ref.	−11.085	15.441	0.4732	−78.026	62.873	0.2153
64<	−29.035	77.925	0.7108	−6.737	19.693	0.7335	Ref.	20.424	26.056	0.4363	−21.240	78.744	0.7883
Gender													
Male	−8.759	88.150	0.9209	19.926	16.443	0.2260	Ref.	−29.603	13.619	0.0301	−56.536	38.816	0.1457
Female	59.246	19.538	0.0025	6.175	5.645	0.2744	Ref.	−8.730	6.527	0.1815	−39.210	25.395	0.1230
HDL cholesterol													
Age													
19~ 44	−3.987	5.399	0.4605	0.135	1.096	0.9021	Ref.	2.554	0.952	0.0075	−0.867	2.954	0.7692
45~ 64	0.799	4.276	0.8518	−1.313	1.360	0.3348	Ref.	−1.687	1.581	0.2868	−5.880	6.408	0.3594
64<	6.579	10.435	0.5309	−1.360	2.688	0.6149	Ref.	3.663	3.507	0.3007	10.076	10.581	0.3450
Gender													
Male	−4.968	6.373	0.4360	−0.661	1.201	0.5824	Ref.	1.028	0.994	0.3017	0.343	2.981	0.9085
Female	1.003	3.743	0.7888	0.050	1.089	0.9634	Ref.	1.908	1.256	0.1292	−3.308	4.865	0.4968
Blood pressure													
Age													
19~ 44	9.081	5.089	0.0747	−0.032	1.029	0.9751	Ref.	0.639	0.893	0.4741	1.560	2.646	0.5556
45~ 64	−4.007	5.626	0.4767	−2.552	1.779	0.1522	Ref.	−3.081	2.075	0.1382	−1.728	8.439	0.8378
64<	−12.387	20.182	0.5417	5.314	5.101	0.3017	Ref.	−2.610	6.749	0.7003	22.083	20.395	0.2833
Gender													
Male	−12.655	7.995	0.1139	2.073	1.497	0.1666	Ref.	−0.438	1.239	0.7239	2.575	3.522	0.4649
Female	4.899	3.870	0.2060	−2.082	1.122	0.0638	Ref.	− 0.001	1.294	0.9996	3.303	5.030	0.5116
Fasting glucose													
Age													
19~ 44	−3.880	7.421	0.6012	1.190	1.494	0.4261	Ref.	1.333	1.299	0.3050	17.139	3.859	<.0001
45~ 64	5.895	5.216	0.2591	1.833	1.648	0.2665	Ref.	−2.049	1.920	0.2865	14.249	7.817	0.0690
64<	13.834	18.440	0.4561	−0.592	4.660	0.8994	Ref.	1.248	6.166	0.8403	3.026	18.633	0.8715
Gender													
Male	0.545	11.505	0.9623	3.968	2.146	0.0649	Ref.	−0.335	1.778	0.8506	21.236	5.066	<.0001
Female	3.278	3.480	0.3466	0.346	1.006	0.7307	Ref.	1.431	1.163	0.2187	5.173	4.524	0.2532

^a^Model is adjusted by age, gender, education level, marital status, household income level, region, occupation, alcohol consumption, smoking, walking activity and stress level

Additional file 1: Table S1 presents the subgroup analysis of the association between the components of metabolic syndrome and TSH levels stratified by age and gender. Additional file 1: Table S2 shows the subgroup analysis of the association between the components of metabolic syndrome and TPOab levels with categorizing FT4 levels stratified by age and gender. In addition, Additional file 1: Table S3 shows the subgroup analysis of the association between the components of metabolic syndrome and TPOab levels with categorizing TSH levels stratified by age and gender. TSH was not significantly associated with other components of metabolic syndrome, except HDL cholesterol. Additional file 1: Table S2 shows that a positive association between TPOab and triglycerides levels is more frequently observed in male with high TPOab titers but with low FT4 levels (β = 136.104, *p* = 0.0062).

## Discussion

The purpose of this study was to identify whether any associations exist between thyroid hormone levels and metabolic syndrome components. Thyroid dysfunction is well known to affect glucose and lipid metabolism; abnormal glucose level and abnormal lipid profile are important factors of metabolic syndrome [17]. In our study, we found that glucose and lipid metabolism were associated with thyroid dysfunction. A significant positive association was observed between fasting glucose and FT4 in patients with high FT4 levels. In terms of lipid metabolism, a negative association between triglycerides and FT4 was observed among people with normal-high or high FT4 levels. In addition, there was a significant positive association between TPOab and triglycerides levels in patients with high thyroid peroxidase levels and with low median FT4 levels.

Thyroid hormones regulate carbohydrate metabolism [18]. They influence the mRNA and protein expression of the glucose transporter 4, AMP-activated protein kinase, and acetyl CoA carboxylase in skeletal muscle [19]. Hyperthyroidism usually occurs when high FT4 levels leads to its increased production and absorption from glycogen, lactic acid, glycerol, and amino acids [20]. Hyperthyroidism can also increase insulin degradation. Which deteriorates blood glucose control [19, 20]. Based on these mechanisms, other studies also showed that glucose levels are high in patients with hyperthyroidism [21]. Foss et al. demonstrated that patients with hyperthyroidism had higher blood glucose levels than the euthyroid participants, indicating increased endogenous glucose production [22]. Moreover, Roubsanthisuk et al. reported that the higher glucose intolerance showed common in hyperthyroidism compare to the normal participants [23]. This could be a major cause of the increased risk for diabetes [23]. Health professionals should conduct follow-up tests of the glucose level of patients with high thyroid hormone levels. Additionally, thyroid hormone levels should be checked regularly in patients with high glucose levels.

A significant negative association between FT4 levels and triglycerides was observed in patients with high-normal or high FT4 levels. To explain this, we presumed that thyroid hormones play a key role in the regulation of enzyme activity during lipoprotein transport [12]. The influence of thyroid function on lipid metabolism involves a pathophysiological process [24]. The negative association observed between triglycerides and thyroid hormones can be explained by the increased removal rate of triglycerides from plasma due to an increase in the activity of hepatic triglyceride lipase [25, 26]. The effects of thyroid hormone on lipid metabolism are well known [26]. Because the lipid profile tends to normalize improperly under high thyroid hormone levels, the lipid profile needs to be followed up after adjusting for thyroid hormone levels [25].

There was a significant positive association between TPOab and triglycerides levels in patients with high thyroid peroxidase levels but with low median FT4 levels. In addition, a positive association was identified between TPOab and triglycerides levels in patients with high thyroid peroxidase levels in the presence of high median TSH levels. The presence of TPOab in the blood indicates a high risk for thyroid disease due to autoimmune disorders [27, 28]. In fact, abnormal TPOab levels were observed in 90% of patients with hypothyroidism and Graves’ disease [27, 28]. Therefore, the positive association between TPOab and triglycerides levels in patients with high thyroid peroxidase levels but low median FT4 levels may be related to hypothyroidism.

Based on the results of the subgroup analysis, a significant negative association was observed between FT4 and triglyceride levels in patients aged 19–44 years; this association was stronger in the 19–44-year-old age group than in the other older age groups. This finding emphasizes the possibility of accumulation of risk through one’s life course. Accumulation of various risks increases owing to illness, health-damaging behaviors, and adverse environmental conditions [29]. Older people may have more risk factors owing to their risk accumulation, and are more likely to have unmeasured risk factors that we could not adjust for [30]. To address this issue, future studies developed to understand the association between thyroid hormones and metabolic syndrome should incorporate a panel design.

This study had some limitations. First, the study was based on a cross-sectional survey. Causality could not be confirmed clearly and only the association could be confirmed. Second, one of the major thyroid hormones, triiodothyronine, was not used in the thyroid function tests. Despite the above limitations, this study also has a few strengths. First, consistent blood tests showed accurate blood TSH levels. Second, most of the previous studies were conducted in Caucasians; therefore, this issue is worth pursuing among Koreans. Finally, this study used the most recent (KNHANES 2013–2015) nationally, multistage, stratified collected data and could therefore be considered to be representative of the Korean population.

## Conclusion

FT4 showed a partially positive association with fasting glucose, and a partially negative association with triglycerides in the general Korean population. Glucose level and lipid profile are metabolic syndrome components; metabolic syndrome is strongly correlated with diseases such as diabetes and cardiovascular disease that are associated with high mortality and morbidity rates. In patients with abnormal thyroid function, follow up tests for metabolic syndrome components during thyroid dysfunction could prevent overlooking metabolic syndrome and prevent related diseases.

## Additional file

Additional file 1: **Table S1**. Subgroup analysis of components of metabolic syndromes with thyroid stimulating hormone levels stratified by age and gender. **Table S2**. Subgroup analysis of components of metabolic syndromes with thyroid peroxidase antibody levels stratified by age and gender. **Table S3**. Subgroup analysis of components of metabolic syndromes with thyroid peroxidase antibody levels stratified by age and gender. (DOCX 88 kb)

## Abbreviations

CI: Confidence interval
FT4: Free thyroxine
KNHANES: Korea National Health and Nutrition Examination Surveys
OR: Odds ratio
TPOab: Thyroid peroxidase antibody
TSH: Thyroid-stimulating hormone
