# Supplementary material for: Association between thyroid hormones and the components of metabolic syndrome
Source: BMC Endocr Disord. 2018 May 21;18:29. doi: 10.1186/s12902-018-0256-0 (PMC5963056; doi:10.1186/s12902-018-0256-0)
Supplement: Supplementary file 1 — Table S1. Subgroup analysis of components of metabolic syndromes with thyroid stimulating hormone levels stratified by age and gender. Table S2. Subgroup analysis of components of metabolic syndromes with thyroid peroxidase antibody levels stratified by age and gender. Table S3. Subgroup analysis of components of metabolic syndromes with thyroid peroxidase antibody levels stratified by age and gender. (DOCX 88 kb) [file 12902_2018_256_MOESM1_ESM.docx]

| **Table S1. Subgroup analysis of components of metabolic syndromes with thyroid stimulating hormone levels stratified by age and gender.** | | | | | | | | | | | | | | | |
| --- | --- | --- | --- | --- | --- | --- | --- | --- | --- | --- | --- | --- | --- | --- | --- |
|  | **Variables** | | **Thyroid stimulating hormone levels** | | | | | | | | | | | | |
|  |  |  |  |  |  |  |  |  |  |  |  |  |  |  |  |
|  |  |  | **Low** | | | **Normal (low tertile)** | | | **Normal (mid tertile)** | **Normal (high tertile)** | | | **High** | | |
|  |  |  |  |  |  |  |  |  |  |  |  |  |  |  |  |
|  |  |  | **β*** | **S.E** | **p-value** | **β*** | **S.E** | **p-value** | **β*** | **β*** | **S.E** | **p-value** | **β*** | **S.E** | **p-value** |
| **Waist circumference** | | |  |  |  |  |  |  |  |  |  |  |  |  |  |
|  | **Age** |  |  |  |  |  |  |  |  |  |  |  |  |  |  |
|  |  | 19~44 | -1.178 | 0.935 | 0.2083 | -0.019 | 0.312 | 0.9504 | Ref. | -0.438 | 0.349 | 0.2105 | 0.225 | 0.512 | 0.6603 |
|  |  | 45~64 | -0.344 | 1.066 | 0.7472 | -0.607 | 0.373 | 0.1040 | Ref. | -0.481 | 0.399 | 0.2283 | -0.022 | 0.605 | 0.9713 |
|  |  | 64< | -2.377 | 2.190 | 0.2821 | -0.214 | 1.059 | 0.8405 | Ref. | 1.775 | 1.119 | 0.1182 | -0.336 | 1.377 | 0.8083 |
|  | **Gender** |  |  |  |  |  |  |  |  |  |  |  |  |  |  |
|  |  | Male | -1.270 | 1.116 | 0.2557 | -0.256 | 0.343 | 0.4553 | Ref. | -0.370 | 0.401 | 0.3567 | -0.089 | 0.605 | 0.8838 |
|  |  | Female | -1.253 | 0.814 | 0.1239 | -0.292 | 0.314 | 0.3518 | Ref. | -0.467 | 0.326 | 0.1528 | -0.242 | 0.461 | 0.5994 |
| **Triglycerides** | | |  |  |  |  |  |  |  |  |  |  |  |  |  |
|  | **Age** |  |  |  |  |  |  |  |  |  |  |  |  |  |  |
|  |  | 19~44 | -28.476 | 28.425 | 0.3167 | -0.953 | 9.485 | 0.9200 | Ref. | -0.244 | 10.617 | 0.9817 | 16.991 | 15.573 | 0.2755 |
|  |  | 45~64 | -24.652 | 40.406 | 0.5421 | 6.410 | 14.134 | 0.6504 | Ref. | 2.643 | 15.119 | 0.8613 | 5.992 | 22.924 | 0.7939 |
|  |  | 64< | 31.006 | 46.855 | 0.5107 | -1.068 | 22.650 | 0.9625 | Ref. | 35.853 | 23.950 | 0.1397 | -7.694 | 29.463 | 0.7949 |
|  | **Gender** |  |  |  |  |  |  |  |  |  |  |  |  |  |  |
|  |  | Male | -33.683 | 45.448 | 0.4589 | 6.836 | 13.974 | 0.6249 | Ref. | 3.819 | 16.323 | 0.8151 | -7.117 | 24.647 | 0.7729 |
|  |  | Female | -8.001 | 16.224 | 0.6221 | -6.967 | 6.257 | 0.2659 | Ref. | 3.860 | 6.501 | 0.5529 | 17.579 | 9.192 | 0.0562 |
| **HDL cholesterol** | | |  |  |  |  |  |  |  |  |  |  |  |  |  |
|  | **Age** |  |  |  |  |  |  |  |  |  |  |  |  |  |  |
|  |  | 19~44 | 8.651 | 2.923 | 0.0032 | -0.075 | 0.987 | 0.9397 | Ref. | 1.501 | 1.111 | 0.1770 | -0.958 | 1.617 | 0.5538 |
|  |  | 45~64 | -1.969 | 4.103 | 0.6316 | -1.207 | 1.448 | 0.4050 | Ref. | -1.548 | 1.543 | 0.3164 | -0.837 | 2.328 | 0.7195 |
|  |  | 64< | 3.258 | 6.354 | 0.6101 | -0.957 | 3.150 | 0.7623 | Ref. | -4.467 | 3.286 | 0.1793 | -5.059 | 3.989 | 0.2099 |
|  | **Gender** |  |  |  |  |  |  |  |  |  |  |  |  |  |  |
|  |  | Male | 7.698 | 3.248 | 0.0181 | -0.417 | 1.013 | 0.6807 | Ref. | 0.911 | 1.183 | 0.4415 | -0.798 | 1.783 | 0.6546 |
|  |  | Female | 3.005 | 3.095 | 0.3320 | -0.379 | 1.207 | 0.7537 | Ref. | -0.466 | 1.251 | 0.7096 | -0.998 | 1.756 | 0.5700 |
| **Blood pressure** | | |  |  |  |  |  |  |  |  |  |  |  |  |  |
|  | **Age** |  |  |  |  |  |  |  |  |  |  |  |  |  |  |
|  |  | 19~44 | 0.972 | 2.845 | 0.7328 | 1.393 | 0.924 | 0.1319 | Ref. | 0.514 | 1.034 | 0.6195 | 1.673 | 1.514 | 0.2695 |
|  |  | 45~64 | 4.763 | 5.397 | 0.3780 | 0.236 | 1.889 | 0.9008 | Ref. | 2.184 | 2.019 | 0.2799 | -1.276 | 3.061 | 0.6771 |
|  |  | 64< | 10.208 | 12.109 | 0.4026 | -5.632 | 5.853 | 0.3399 | Ref. | 8.310 | 6.189 | 0.1845 | 5.591 | 7.614 | 0.4657 |
|  | **Gender** |  |  |  |  |  |  |  |  |  |  |  |  |  |  |
|  |  | Male | 3.462 | 4.097 | 0.3984 | 0.859 | 1.262 | 0.4966 | Ref. | 2.249 | 1.475 | 0.1278 | -0.584 | 2.222 | 0.7926 |
|  |  | Female | 2.160 | 3.298 | 0.5128 | 0.897 | 1.240 | 0.4694 | Ref. | 0.875 | 1.286 | 0.4963 | 1.434 | 1.818 | 0.4305 |
| **Fasting glucose** | | |  |  |  |  |  |  |  |  |  |  |  |  |  |
|  | **Age** |  |  |  |  |  |  |  |  |  |  |  |  |  |  |
|  |  | 19~44 | -0.853 | 4.065 | 0.8339 | 2.159 | 1.356 | 0.1119 | Ref. | 0.613 | 1.518 | 0.6866 | -0.950 | 2.227 | 0.6696 |
|  |  | 45~64 | 3.067 | 5.039 | 0.5430 | 1.439 | 1.763 | 0.4147 | Ref. | -0.470 | 1.886 | 0.8034 | -0.504 | 2.859 | 0.8603 |
|  |  | 64< | -12.046 | 10.991 | 0.2776 | -5.523 | 5.313 | 0.3028 | Ref. | 3.245 | 5.618 | 0.5658 | -2.496 | 6.912 | 0.7193 |
|  | **Gender** |  |  |  |  |  |  |  |  |  |  |  |  |  |  |
|  |  | Male | -2.724 | 5.950 | 0.6472 | 3.318 | 1.829 | 0.0702 | Ref. | 2.967 | 2.137 | 0.1655 | -2.774 | 3.226 | 0.3902 |
|  |  | Female | 1.822 | 2.874 | 0.5265 | 0.225 | 1.109 | 0.8391 | Ref. | -1.197 | 1.152 | 0.2989 | -0.665 | 1.628 | 0.6832 |

| **Table S2. Subgroup analysis of components of metabolic syndromes with thyroid peroxidase antibody levels stratified by age and gender.** | | | | | | | | | |
| --- | --- | --- | --- | --- | --- | --- | --- | --- | --- |
|  | **Variables** | | **Thyroid peroxidase antibody** | | | | | | |
|  |  |  |  |  |  |  |  |  |  |
|  |  |  | **Normal** | **High (under low FT4)** | | | **High (under high FT4)** | | |
|  |  |  |  |  |  |  |  |  |  |
|  |  |  | **β*** | **β*** | **S.E** | **p-value** | **β*** | **S.E** | **p-value** |
| **Waist circumference** | | |  |  |  |  |  |  |  |
|  | **Age** |  |  |  |  |  |  |  |  |
|  |  | 19~44 | Ref. | -0.508 | 0.929 | 0.5847 | -1.489 | 0.826 | 0.0719 |
|  |  | 45~64 | Ref. | 0.628 | 0.579 | 0.2783 | 0.764 | 0.814 | 0.3489 |
|  |  | 64< | Ref. | 5.313 | 2.723 | 0.0556 | -2.355 | 1.600 | 0.1461 |
|  | **Gender** |  |  |  |  |  |  |  |  |
|  |  | Male | Ref. | -0.134 | 1.223 | 0.9127 | -1.372 | 0.880 | 0.1197 |
|  |  | Female | Ref. | 0.179 | 0.519 | 0.7300 | -0.035 | 0.695 | 0.9598 |
| **Triglycerides** | | |  |  |  |  |  |  |  |
|  | **Age** |  |  |  |  |  |  |  |  |
|  |  | 19~44 | Ref. | 48.165 | 28.212 | 0.0881 | -6.683 | 25.100 | 0.7901 |
|  |  | 45~64 | Ref. | 19.386 | 21.903 | 0.3766 | 1.954 | 30.829 | 0.9495 |
|  |  | 64< | Ref. | 105.206 | 58.306 | 0.0761 | -6.732 | 34.253 | 0.8448 |
|  | **Gender** |  |  |  |  |  |  |  |  |
|  |  | Male | Ref. | 136.104 | 49.548 | 0.0062 | -30.771 | 35.655 | 0.3885 |
|  |  | Female | Ref. | 11.099 | 10.353 | 0.2841 | 17.220 | 13.872 | 0.2149 |
| **HDL cholesterol** | | |  |  |  |  |  |  |  |
|  | **Age** |  |  |  |  |  |  |  |  |
|  |  | 19~44 | Ref. | -4.475 | 2.917 | 0.1254 | 1.840 | 2.661 | 0.4895 |
|  |  | 45~64 | Ref. | -0.291 | 2.296 | 0.8994 | -0.398 | 3.136 | 0.8992 |
|  |  | 64< | Ref. | 0.455 | 8.226 | 0.9560 | -1.498 | 4.782 | 0.7552 |
|  | **Gender** |  |  |  |  |  |  |  |  |
|  |  | Male | Ref. | -3.216 | 3.576 | 0.3688 | 2.291 | 2.650 | 0.3878 |
|  |  | Female | Ref. | -2.240 | 2.006 | 0.2644 | -1.749 | 2.635 | 0.5072 |
| **Blood pressure** | | |  |  |  |  |  |  |  |
|  | **Age** |  |  |  |  |  |  |  |  |
|  |  | 19~44 | Ref. | 4.279 | 2.741 | 0.1189 | 2.732 | 2.439 | 0.2629 |
|  |  | 45~64 | Ref. | -4.151 | 2.919 | 0.1557 | 5.815 | 4.110 | 0.1578 |
|  |  | 64< | Ref. | -3.505 | 15.734 | 0.8245 | 2.910 | 9.243 | 0.7540 |
|  | **Gender** |  |  |  |  |  |  |  |  |
|  |  | Male | Ref. | -7.130 | 4.494 | 0.1131 | 0.820 | 3.233 | 0.7999 |
|  |  | Female | Ref. | 0.152 | 2.030 | 0.9403 | 7.391 | 2.720 | 0.0067 |
| **Fasting glucose** | | |  |  |  |  |  |  |  |
|  | **Age** |  |  |  |  |  |  |  |  |
|  |  | 19~44 | Ref. | -1.617 | 4.042 | 0.6893 | -3.734 | 3.596 | 0.2993 |
|  |  | 45~64 | Ref. | -3.841 | 2.730 | 0.1601 | 0.826 | 3.843 | 0.8299 |
|  |  | 64< | Ref. | -13.962 | 13.893 | 0.3189 | 6.448 | 8.161 | 0.4326 |
|  | **Gender** |  |  |  |  |  |  |  |  |
|  |  | Male | Ref. | -1.366 | 6.555 | 0.8349 | -1.759 | 4.717 | 0.7094 |
|  |  | Female | Ref. | -3.170 | 1.826 | 0.0831 | -1.016 | 2.447 | 0.6782 |

| **Table S3. Subgroup analysis of components of metabolic syndromes with thyroid peroxidase antibody levels stratified by age and gender.** | | | | | | | | | |
| --- | --- | --- | --- | --- | --- | --- | --- | --- | --- |
|  | **Variables** | | **Thyroid peroxidase antibody** | | | | | | |
|  |  |  |  |  |  |  |  |  |  |
|  |  |  | **Normal** | **High (under low TSH)** | | | **High (under high TSH)** | | |
|  |  |  |  |  |  |  |  |  |  |
|  |  |  | **β*** | **β*** | **S.E** | **p-value** | **β*** | **S.E** | **p-value** |
| **Waist circumference** | | |  |  |  |  |  |  |  |
|  | **Age** |  |  |  |  |  |  |  |  |
|  |  | 19~44 | Ref. | -1.423 | 0.950 | 0.1348 | -0.792 | 0.813 | 0.3304 |
|  |  | 45~64 | Ref. | 0.898 | 0.795 | 0.2593 | 0.558 | 0.579 | 0.3353 |
|  |  | 64< | Ref. | 0.125 | 1.843 | 0.9461 | -1.202 | 2.184 | 0.5841 |
|  | **Gender** |  |  |  |  |  |  |  |  |
|  |  | Male | Ref. | -0.399 | 1.108 | 0.7193 | -1.345 | 0.930 | 0.1488 |
|  |  | Female | Ref. | -0.239 | 0.659 | 0.7176 | 0.321 | 0.532 | 0.5462 |
| **Triglycerides** | | |  |  |  |  |  |  |  |
|  | **Age** |  |  |  |  |  |  |  |  |
|  |  | 19~44 | Ref. | -11.835 | 28.868 | 0.6819 | 38.718 | 24.695 | 0.1173 |
|  |  | 45~64 | Ref. | -15.180 | 30.068 | 0.6139 | 28.246 | 21.895 | 0.1977 |
|  |  | 64< | Ref. | 49.180 | 38.143 | 0.2021 | -18.282 | 45.205 | 0.6873 |
|  | **Gender** |  |  |  |  |  |  |  |  |
|  |  | Male | Ref. | -24.157 | 45.077 | 0.5922 | 59.179 | 37.835 | 0.1183 |
|  |  | Female | Ref. | 4.304 | 13.163 | 0.7438 | 18.906 | 10.622 | 0.0755 |
| **HDL cholesterol** | | |  |  |  |  |  |  |  |
|  | **Age** |  |  |  |  |  |  |  |  |
|  |  | 19~44 | Ref. | 0.370 | 2.988 | 0.9016 | -2.078 | 2.617 | 0.4273 |
|  |  | 45~64 | Ref. | -1.172 | 3.166 | 0.7113 | 0.085 | 2.267 | 0.9702 |
|  |  | 64< | Ref. | 0.252 | 5.265 | 0.9621 | -2.855 | 6.261 | 0.6501 |
|  | **Gender** |  |  |  |  |  |  |  |  |
|  |  | Male | Ref. | -1.324 | 3.242 | 0.6832 | 1.639 | 2.816 | 0.5607 |
|  |  | Female | Ref. | -2.252 | 2.552 | 0.3778 | -1.946 | 2.041 | 0.3408 |
| **Blood pressure** | | |  |  |  |  |  |  |  |
|  | **Age** |  |  |  |  |  |  |  |  |
|  |  | 19~44 | Ref. | 0.472 | 2.801 | 0.8663 | 5.548 | 2.397 | 0.0209 |
|  |  | 45~64 | Ref. | -1.118 | 4.032 | 0.7818 | -0.796 | 2.936 | 0.7863 |
|  |  | 64< | Ref. | -5.510 | 10.091 | 0.5870 | 11.082 | 11.960 | 0.3578 |
|  | **Gender** |  |  |  |  |  |  |  |  |
|  |  | Male | Ref. | -4.075 | 4.075 | 0.3177 | -0.292 | 3.421 | 0.9321 |
|  |  | Female | Ref. | 1.208 | 2.590 | 0.6410 | 3.623 | 2.090 | 0.0834 |
| **Fasting glucose** | | |  |  |  |  |  |  |  |
|  | **Age** |  |  |  |  |  |  |  |  |
|  |  | 19~44 | Ref. | -4.342 | 4.135 | 0.2940 | -1.685 | 3.537 | 0.6339 |
|  |  | 45~64 | Ref. | -0.464 | 3.756 | 0.9016 | -3.254 | 2.735 | 0.2347 |
|  |  | 64< | Ref. | 4.217 | 9.087 | 0.6443 | -2.913 | 10.770 | 0.7877 |
|  | **Gender** |  |  |  |  |  |  |  |  |
|  |  | Male | Ref. | 1.935 | 5.935 | 0.7445 | -4.095 | 4.982 | 0.4114 |
|  |  | Female | Ref. | -3.576 | 2.323 | 0.1242 | -1.681 | 1.875 | 0.3704 |
